# Functional Covariance Connectivity of Gray and White Matter in Olfactory-Related Brain Regions in Parkinson’s Disease

**DOI:** 10.3389/fnins.2022.853061

**Published:** 2022-03-04

**Authors:** Yiqing Wang, Hongyu Wei, Shouyun Du, Hongjie Yan, Xiaojing Li, Yijie Wu, Jianbing Zhu, Yi Wang, Zenglin Cai, Nizhuan Wang

**Affiliations:** ^1^Department of Neurology, The Affiliated Suzhou Science and Technology Town Hospital of Nanjing Medical University, Suzhou, China; ^2^Department of Neurology, Gusu School, Nanjing Medical University, Suzhou, China; ^3^Department of Neurology, Guanyun People’s Hospital, Lianyungang, China; ^4^Department of Neurology, Affiliated Lianyungang Hospital of Xuzhou Medical University, Lianyungang, China; ^5^Department of Radiology, The Affiliated Suzhou Science and Technology Town Hospital of Nanjing Medical University, Suzhou, China; ^6^School of Biomedical Engineering, ShanghaiTech University, Shanghai, China

**Keywords:** Parkinson’s disease, resting state fMRI, gray matter, white matter, hyposmia, functional covariance connectivity

## Abstract

Before the onset of motor symptoms, Parkinson’s disease (PD) involves dysfunction of the anterior olfactory nucleus and olfactory bulb, causing olfactory disturbance, commonly resulting in hyposmia in the early stages of PD. Accumulating evidence has shown that blood oxygen level dependent (BOLD) signals in white matter are altered by olfactory disorders and related stimuli, and the signal changes in brain white matter pathways show a certain degree of specificity, which can reflect changes of early olfactory dysfunction in Parkinson’s disease. In this study, we apply the functional covariance connectivity (FCC) method to decode FCC of gray and white matter in olfactory-related brain regions in Parkinson’s disease. Our results show that the dorsolateral prefrontal, anterior entorhinal cortex and fronto-orbital cortices in the gray matter have abnormal connectivity with the posterior corona radiata and superior corona radiata in white matter in patients with Parkinson’s hyposmia. The functional covariance connection strength (FCS) of the right dorsolateral prefrontal cortex and white matter, and the covariance connection strength of the left superior corona radiata and gray matter function have potential diagnostic value. These results demonstrate that alterations in FCC of gray and white matter in olfactory-related brain regions can reflect the change of olfactory function in the early stages of Parkinson’s disease, indicating that it could be a potential neuroimaging marker for early diagnosis.

## Introduction

Parkinson’s disease (PD) is a progressive neurodegenerative disease that often occurs in middle to old age, and its prevalence gradually increases with aging (Kalia and Lang, 2015; Abeliovich and Gitler, 2016). PD usually undergoes 5–20 years of pre-motor symptoms state before typical motor symptoms, including the early physiological changes of PD, pre-clinical, pre-motor symptoms, and pre-diagnosis of PD (Siderowf and Stern, 2006). Braak et al. (2003) divided PD into six pathology-based stages based on the order of appearance of Lewy bodies, where Stage 1 includes degeneration of the olfactory bulb and pre-olfactory nucleus, leading to clinical olfactory disorders. Clinically, before the onset of motor symptoms, PD will involve the pre-olfactory nucleus and the olfactory bulb and cause olfactory disorders, and hyposmia is a common clinical manifestation in the early stage of PD (Ansari and Johnson, 1975). The reported prevalence of hyposmia in early Parkinson’s disease is up to 90%, making it a potential biomarker of the disease (Xiao et al., 2014), and the hyposmia gradually worsens as the disease progresses after entering the motor-symptom stage (Schapira et al., 2017). It has been accepted as one of the supportive diagnoses of Parkinson’s disease (Postuma et al., 2015) and is considered to be a reliable disease marker (Sui et al., 2019). In a recent meta-analysis (Sui et al., 2019), hyposmia associated with 3.84-fold risk of Parkinson’s disease progression (pooled relative risk: 3.84, 95% CI 2.12-6.95). Therefore, further exploration of Parkinson’s disease with hyposmia can help improve the early diagnosis of Parkinson’s disease, so that patients can receive access to treatment earlier and improve quality of life.

As researchers pay more attention to the abnormal olfactory function associated with PD, there are increasing amounts of Magnetic Resonance Imaging (MRI) studies on PD with hyposmia, including the study of structural changes in the olfactory pathway and MRI functional imaging studies. Wang et al. (2011) used high-resolution magnetic resonance imaging to scan the olfactory bulb on the coronal plane and measure its volume. They found that the volume of the olfactory bulb in PD patients was smaller than in the normal control group, and was correlated with the results of olfactory detection. Scherfler et al. (2006) used diffusion weighted imaging (DWI) imaging technology combined with SPM to calculate Trace (D) in the area near the olfactory tract, and found that the Trace (D) value of the area near the olfactory tract in early PD patients was significantly higher than in the control group. This result is consistent with the early neuropathological changes of the anterior olfactory structure reported in PD patients (Braak et al., 2006), indicating that hyposmia may be related to the destruction of the olfactory tract structure. Westermann et al. (2008) used the blood-oxygen-level-dependent (BOLD) functional magnetic resonance imaging (fMRI) technology to study the neurological function of the brain related areas that process olfactory information in PD patients in the early clinical stage, and found that the neuronal activities of the hippocampus, parahippocampal gyrus, and amygdala of PD patients were significantly reduced compared to healthy people, indicating that impaired hyposmia in PD patients is related to abnormal changes in the central structure related to olfaction.

Although some progress has been made in the research of Parkinson’s disease brain structure and functional magnetic resonance in recent years, researchers have paid less attention to the changes in the connectivity of olfactory-related white matter and gray matter. More and more evidence shows that the BOLD signal in white matter undergoes stimulus-related synchronous changes after olfactory and its related stimuli, but there are different degrees of time delays, and the signal changes in the white matter pathways of the brain show a certain degree of specificity (Ding et al., 2018). With regard to the BOLD signal in gray matter, the amplitude of the low frequency fluctuation (ALFF) or fractional ALFF (fALFF) are effective measures of resting state BOLD signal changes (Zang et al., 2007; Zou et al., 2008), where can extract the spontaneous brain activity. The ALFF index is stable, reliable and can be used to observe intrinsic or spontaneous brain activity in neurodegenerative diseases such as Parkinson’s disease (Wang et al., 2018). Based on the above evidence, we believe that fMRI olfactory-related gray/white matter information in Parkinson’s patients is specific and can reflect the early changes in their olfactory function. Therefore, in this study we selected Parkinson’s patients with hyposmia, and analyzed the differences between Parkinson’s patients with hyposmia and normal subjects. The functional covariance connectivity (FCC) between the white and gray matter voxels was constructed based on ALFF maps (Chen et al., 2021) and explored its value as a potential neuroimaging marker for early diagnosis.

## Materials and Methods

### Participants

A publicly available dataset from the OpenfMRI database was used in this study. The login number is ds000245,^1^ and detailed demographic data can be found in the original article (Yoneyama et al., 2018). The data of 15 PD patients with normal cognitive function but with hyposmia Parkinson’s Disease with Hyposmia (PDH group) and 15 with mild (or without) hyposmia (PDM group) were included. The inclusion criteria were as follows: (1) patients diagnosed with PD according to the United Kingdom Brain Database criteria; (2) no other neurological or psychiatric disorders; (3) focal deep white matter lesions Fazekas classification ≤ 2 score; and (4) no family history of PD. An additional 15 subjects without neurological disease, with normal cognitive function and without olfactory dysfunction served as healthy controls (HC group). Written informed consent was obtained from all participants. The study was approved by the Ethics Committee of the Nagoya University Graduate School of Medicine. Our previous analysis of the FCC analysis of gray and white matter voxels showed that there was no significantly statistical difference in the indicators between the PDM group and PDH or HC groups, thus the data of the PDM group was not included in our later analysis.

### Olfactory and Cognitive Assessment

The odor recognition ability of all participants was tested using the Japanese Odor Stick Identification Test (OSIT-J; Daiichi Yakuhin, Co., Ltd., Tokyo, Japan) (Saito et al., 2006), and their cognitive function was assessed using the revised Addenbrooke’s Cognitive Examination Scale (Addenbrooke’s Cognitive Examination Revised, ACE-R) (Mioshi et al., 2006). The OSIT -J consists of 12 odors familiar to Japanese people and is widely used to assess olfactory function in PD patients. The ACE-R consists of assessments of six cognitive domains (orientation, attention, memory, verbal fluency, language and visuospatial ability) and can be used to diagnose dementia accompanying PD patients. Patients with OSIT-J scores > 4 and ACE-R scores ≤ 88 were excluded.

### Magnetic Resonance Imaging Acquisition

Participants underwent MRI scans with eyes closed while awake, using a 3.0 T scanner (Siemens, Erlangen, Germany) with a 32-channel coil at the Center for Brain and Mind, Nagoya University. High-resolution 3D T1-weighted images were obtained with the following parameters: repetition time (TR) = 2,500 ms, echo time (TE) = 2.48 ms, thickness = 1 mm, 192 sagittal slices, field of view (FOV) = 256 × 256 mm^2^, matrix size = 256 × 256. Resting-state functional MRI images were acquired with the following parameters: *TR* = 2.5 s, *TE* = 30 ms, 39 transverse slices, layer spacing = 0.5 mm, thickness = 3 mm, FOV = 192 mm, matrix size = 64 × 64, flip angle (FA) = 80 degrees. T1-weighted images Total scan time was 349 s. The total resting-state functional MRI images scanning time was 8 min.

### Resting-State Functional Magnetic Resonance Imaging Data Preprocessing

**FIGURE 1:**
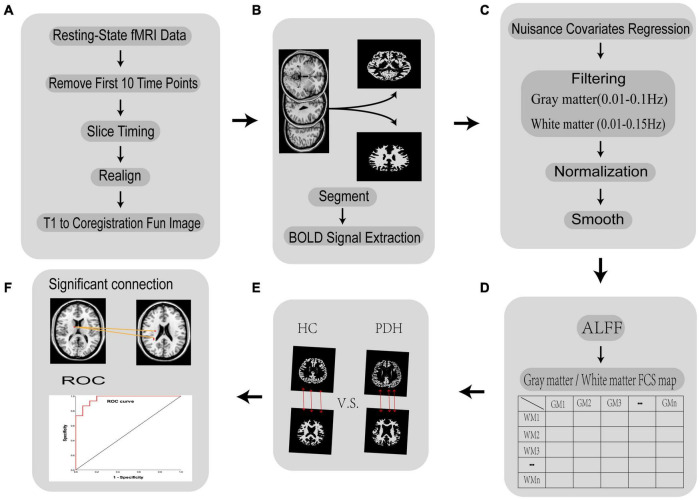
Flow chart of the fMRI data processing in FCC analysis. **(A)** Removal of the first 10 time points, slice time correction, head movement correction, and individual image registration of functional images; **(B)** white matter-gray matter segmentation and BOLD signal extraction; **(C)** remove irrelevant noise, filter the BOLD signal of white matter and gray matter separately, normalize brain volumes to MNI space, smooth spatial brain volumes; **(D)** calculate ALFF and FCS; **(E)** conduct hypothesis testing and multiple comparisons for PDH and HC groups; **(F)** obtain meaningful brain areas and ROC curves.

All resting-state fMRI data were pre-processed using the DPABI toolbox v5.1^2^ in the following steps, which are summarized in Figure 1 (Yan et al., 2016). (1) Remove the first 10 time points. Changes in the initial gradient magnetic field strength and subjects’ adaptation to the environment affect the quality of the data. Therefore, the first 10 time points of each acquired data were removed. (2) Slice timing correction: correction for differences in acquisition time of slice-like image data. (3) Head motion realignment: The subject has head movement during the examination, which will increase the noise of the imaging. The algorithm was used to correct the results. (4) Individual fMRI images were registered to individual structural images. In an fMRI image, each 3D location is a functional time series rather than a gray scale value, so it can be viewed as a four-dimensional image. fMRI images do not have clear visual boundaries, anatomical structures, and it is usually impossible to visually define similarities or differences between images. T1 structural images, on the other hand, have a higher spatial resolution and contain more detailed information about the structure. To facilitate the exploration of the specific locations where the human brain controls a particular function, we aligned the fMRI images to the structural images. (5) Image segmentation: in addition to the various functional brain regions, the MRI scanned images also contain images of structures that are not related to functional brain connectivity, such as ventricles, brain pools, cerebrospinal fluid, skull, scalp, subcutaneous fat, and other structures. The signals from these structural regions that are not related to functional brain connectivity were segmented and removed. Each individual subject creates a mask of gray and white matter, this process was implemented with the assistance of SPM12.^3^ The default method selected for this study is called “New Segment+DARTEL” in SPM8. For each voxel, we defined it as gray matter, white matter, or cerebrospinal fluid (CSF) according to the maximum probability of the three segmented images. (6) Groups of gray and white matter masks were created: for each voxel, a voxel was defined as gray or white matter if it was present in more than 60% of the subjects’ gray or white matter masks. To avoid confusion of signals between gray and white matter, voxels near the boundary between gray and white matter were excluded from the gray or white matter mask. In this study, the group mask was obtained based on the DPABI quality control algorithm. (7) Extraction of gray matter and white matter fMRI image signals: we extracted the image signals by the DPABI image operator equation, which has the expression g1.*To4D(i1,n). Here g1 is the fMRI data, i1 is the group mask and n is the time point. (8) Nuisance covariates regression and adjustment of head movement parameters. To avoid eliminating the signal of interest, we removed only the CSF noise signal and retained the white matter and whole-brain signal. The Friston 24 parameter model was selected for this study to adjust the head movement parameters. (9) Filtering: We used a band-pass filter for filtering to reduce non-neuronal interference to the BOLD signal on the detrended time series. Peer et al. (2017) found that gray and white matter differ in their signals in different frequency domains. The filtered band for gray matter fMRI image signals was 0.01–0.1 Hz, while the filtered band for white matter fMRI image signals was 0.01–0.15 Hz. (10) Spatial alignment: there are individual differences in human brain structure. In order for comparisons of functional signals between subjects to accurately locate brain regions, individual brains are usually normalized to a uniform spatial template. In this study, the image data were resampled and normalized to Montreal Neurological Institute (MNI) space at a voxel size of 4 × 4 × 4 mm^3^. (11) Spatial smoothing: to reduce the errors arising from spatial normalization and to remove random noise from the images, the fMRI images were smoothed with a 4 × 4 × 4 mm^3^ Gaussian kernel function in this study.

### Calculating Functional Covariance Connection Strength

(1) To calculate the ALFF value of each voxel, we used the DPABI default method of calculating the ALFF. In brief the steps are as follows: first the Fourier transform is obtained for low frequency fluctuations, the square root of the power spectrum is obtained, summed, and then divided by the number of time points for averaging, before being finally normalized using the whole-brain average ALFF.
(2) Two ALFF images were obtained for each subject (one gray and one white matter ALFF image). Because the group mask is constructed from individual masks based on probability, and the individual masks are heterogeneous, there may be a mixture of gray matter group masks and white matter group masks at actual anatomical locations. Therefore, we used the and white matter masks in MRIcroN^4^ software, and removed the mixed signals from the images using matrix dot product operations in MATLAB.^5^ All image operations are on a space of 47 × 56 × 46 mm^3^.
(3) Functional covariance connection strength (FCS) was calculated for each subject.
  Drawing on the definition of structural covariance linkage (Eisenberg et al., 2015), the functional covariance connection (FCC) was defined as the Pearson correlation coefficient between the ALFF values of two voxels across the subjects, and was calculated as follows:
  $$\text{r}=\frac{1}{N-1}\sum_{\text{i}=1}^{\text{N}}\left(\frac{{\text{X}}_{\text{i}}-\bar{\text{X}}}{{\text{S}}_{\text{X}}}\right)\left(\frac{{\text{Y}}_{\text{i}}-\bar{\text{Y}}}{{\text{S}}_{\text{Y}}}\right)=\frac{1}{N-1}\sum_{\text{i}=1}^{\text{N}}{\text{Z}}_{\text{Xi}}{\text{Z}}_{\text{Yi}},$$
  where N is the number of subjects, $\bar{\text{X}}$ and $\bar{\text{Y}}$ are the mean values of the data X and Y, and S is the sample standard deviation. The Pearson correlation coefficient (r) between the data series X and Y can be written as the normalized inner product of standard scores (z-scores). We use the product (Z_Xi_Z_Yi_) of the z-score of each individual (i) as a measure of the strength of the individual’s functional connection. The product of the standard scores of each pair of voxels is the FCS. The FCS can be used to measure the strength of the functional covariance of each pair of voxels (Eisenberg et al., 2015; Chen et al., 2021).
(4) Regions of interest (ROI) were obtained. For each subject, we obtained a voxel-based 28,467 × 648 functional covariance connection matrix (28,467 represents the number of gray matter voxels, and 648 represents the number of white matter voxels). To test whether each gray matter-white matter connection differed between the PDH and HC groups, this study used a *t*-test to compare the functional covariance contribution values. *P* < 0.000005 was considered significant. Areas with differences in gray matter-white matter connectivity areas compared to controls were considered as regions of interest. The BrainNet Viewer was used to show the significant FCC between the voxels from the gray and white matters (Xia et al., 2013).

### Obtaining Significantly Different Brain Regions

We recreated the regions with differential white matter-gray matter connectivity as two masks: white matter and gray matter. These two masks served as ROIs. For each subject, the pre-processed ALFF data images were calculated with the ROI to obtain white matter and gray matter ALFF data images, using the same computational principle as described above for removing mixed signals. We calculated the FCS between the gray matter and white matter for each subject to obtain a 19 × 16 FCC matrix. (19 represents the number of gray matter voxels and 16 represents the number of white matter voxels). For the white matter-gray matter connection, we performed the *t*-test, and searched for brain regions that had significant differences in the contribution of functional covariance between the PDH group and the HC group (*p* < 0.05, FDR correction).

### Drawing the Receiver Operating Characteristic Curve

We used the connection strength values of functional covariance connections between gray and white matter voxels to predict the probability of normal control or Parkinson’s disease. Specifically, for a given gray matter voxel, we calculated the average FCS value for that gray matter voxel vs. the 16 white matter voxels, and for a given white matter voxel, we calculated the average FCS value for that white matter voxel vs. 19 gray matter voxels. We analyzed the average FCS value using the first step method in the binary logistic regression analysis method in SPSS software (IBM Corp. Released 2019, IBM SPSS Statistics for Windows, version 26.0). The 19 gray matter voxels and 16 white matter voxels with good PD prediction effect were screened. If the variables entered the equation were obtained, the Receiver Operating Characteristic (ROC) curve was drawn and the area under the curve (AUC) was calculated.

## Results

### Abnormal Functional Covariance Connections for Healthy Controls and PDH Groups

**FIGURE 2:**
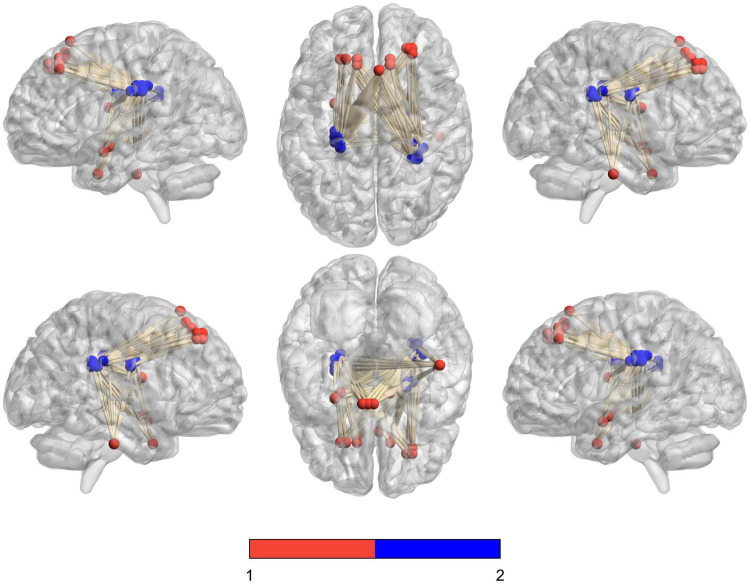
Schematic diagram of the abnormal functional covariance connections between 19 gray matter voxels and 16 white matter voxels identified by FCC analysis between the PDH and HC groups. The red balls represent gray matter voxels, and the blue balls are the white matter voxels.

**FIGURE 3:**
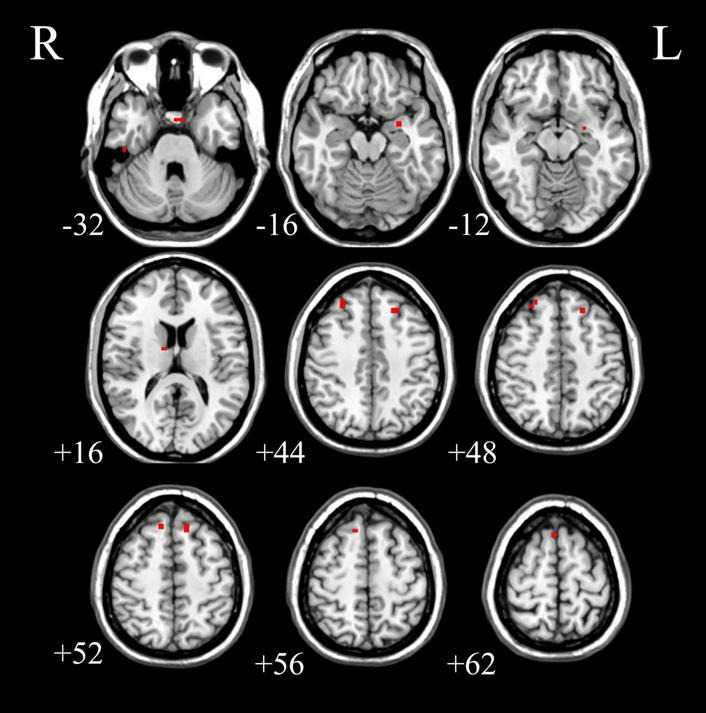
The gray matter areas in abnormal functional covariance connections. Areas marked in red are brain gray matter areas where FCS is statistically different between the PDH and HC groups (two sample *t*-test, *p* < 0.05, FDR correction).

**FIGURE 4:**
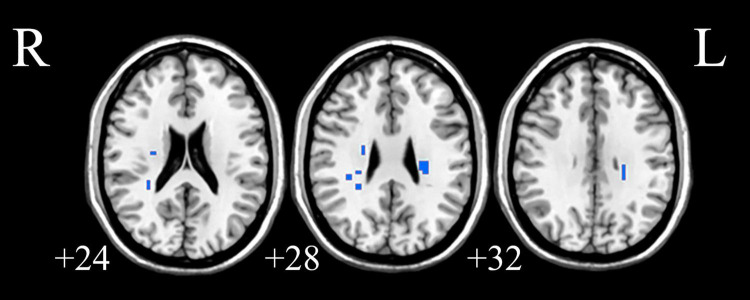
The white matter areas in abnormal functional covariance connections. Areas marked in blue are brain white matter areas where FCS is statistically different between the PDH and HC groups (two sample *t*-test, *p* < 0.05, FDR correction).

We analyzed the voxel-based 28,467 × 648 FCC matrix, where 28,467 represented the number of gray matter voxels, and 648 represented the number of white matter voxels. Then, we identified 19 × 16 significantly different functional covariance connections by FCC analysis in the PDH group compared to the control group, and the corresponding results were shown in Figure 2. Furthermore, there were differences between the FCS of multiple brain gray matter regions between the HC and PDH groups. These areas were located mainly dorsolateral prefrontal cortex, frontal eye field, and anterior entorhinal cortex (please see Table 1 and Figure 3 for details). Finally, there were also differences in FCS in many white matter areas between the HC and PDH groups. These areas were mainly located in the superior corona radiata and posterior corona radiata (please refer to Table 2 and Figure 4 for details).

**TABLE 1:** Gray matter brain areas with significant differences from the PDH group and control group based on Brodmann atlas.

Coordinates			Brodmann areas
X	Y	Z	
−20	32	44	Dorsolateral prefrontal cortex (Brodmann 9)
−24	32	44	
28	36	44	
28	40	44	
−20	32	48	
28	36	48	
24	40	48	
12	32	52	
−28	0	−16	Anterior entorhinal cortex (Brodmann 34)
−24	−4	−12	
48	−24	−32	Unclassified
0	4	−32	
−4	4	−32	
−8	4	−32	
12	−4	16	
−12	32	52	Part of frontal eye field (Brodmann 8)
12	28	56	
4	24	64	
−12	28	52	

*X, Y, Z are the spatial coordinates in MNI space.*

**TABLE 2:** White matter brain areas with significant differences from the PDH group and control group based on JHU-White Matter atlas.

Coordinates			Brain regions
X	Y	Z	
28	−12	24	Superior corona radiata
36	−32	28	
−24	−20	28	
−28	−20	28	
24	−12	28	
24	−8	28	
−24	−24	32	
32	−40	24	Unclassified
32	−36	24	
28	−40	28	Posterior corona radiata
28	−28	28	
−28	−28	28	
−24	−24	28	
−28	−24	28	
−24	−32	32	
−24	−28	32	

*X, Y, Z are the spatial coordinates in MNI space.*

### Functional Covariance Connection Strength Characteristics of the Abnormal Gray/White Matter

**FIGURE 5:**
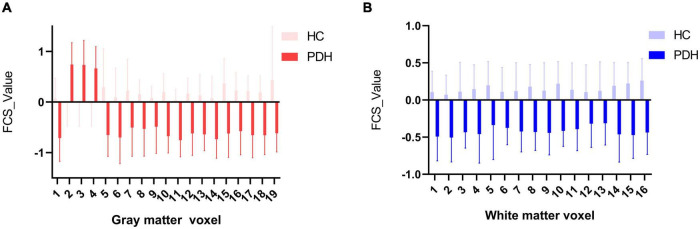
**(A)** Comparison of HC and PDH in average FCS of gray matter voxels; **(B)** comparison of HC and PDH in average FCS of white matter voxels.

In this part, we have explored the FCS characteristics of the gray/white matter voxels involved in the abnormal functional covariance connections. In terms of the 19 gray matter voxels, we found some brain regions with lower FCS values than the control group, as shown in Figure 5A. The white matter part of the 19 × 16 white-gray matter functionally connected is composed of 16 white matter voxels. We found that among the 16 white matter voxels, the FCS value of the brain area was lower than that of the control group (please see Figure 5B for details).

### ROC Curve Results

**FIGURE 6:**
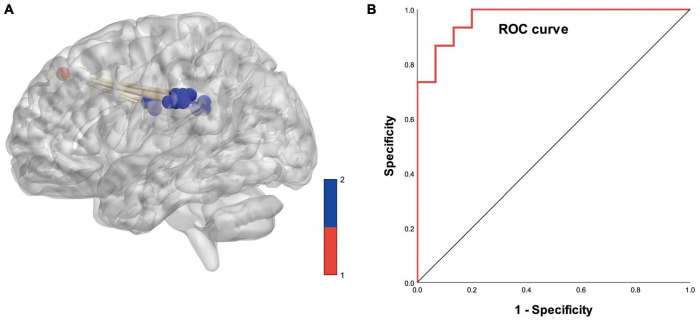
**(A)** Diagram of functional covariance connections between the right dorsolateral prefrontal cortex and white matter. Red spheres represent gray matter voxels in the right dorsolateral prefrontal cortex, and blue spheres represent white matter voxels. **(B)** ROC curve based on the connections between the right dorsolateral prefrontal cortex and white matter voxels (area under the curve is 0.969).

Based on logistic analysis of FCS values across 19 gray matter voxels, we found that FCS values in the dorsolateral prefrontal cortex of the right cerebral hemisphere were an independent risk factor for PD. We analyzed the reliability of the model prediction results. The AUC value of the model was 0.969; the significance level of AUC was statistically significant (*p* < 0.05) (details are shown in Figure 6).

**FIGURE 7:**
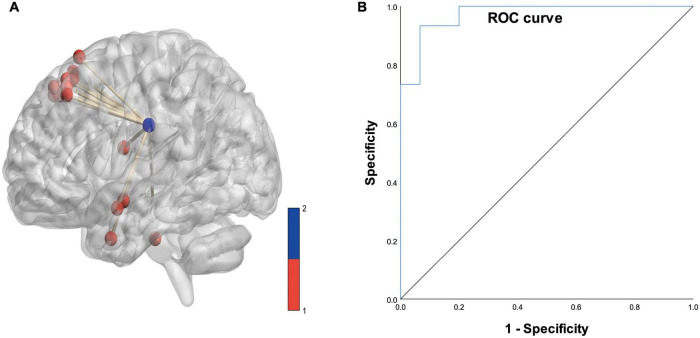
**(A)** Diagram of the functional covariance connections between the left superior corona radiata and gray matter voxels. Red spheres represent gray matter voxels, and the blue spheres represent the left superior corona radiata. **(B)** ROC curve based on the connections between the left superior corona radiata and the gray matter voxels (area under the curve is 0.973).

Based on logistic analysis of 16 white matter voxel FCS values, we found that the FCS value of the superior corona radiata of the left cerebral hemisphere was an independent risk factor for Parkinson’s disease. The ROC curve analysis showed that the AUC value of the predictive model was 0.973 (details are shown in Figure 7).

## Discussion

Parkinson’s disease is one of the most common neurodegenerative diseases, and is mainly characterized by the progressive loss of dopaminergic neurons in the substantia nigra. In 2016, 6.1 million people were reported to have been diagnosed with PD worldwide (GBD 2016 Neurology Collaborators, 2019; Dong et al., 2022). Although treatments for PD can be used to ameliorate certain symptoms, they cannot slow down or halt the disease progression, and as the disease progresses the clinical benefits of various treatments gradually fade, while side effects gradually appear and increase (Olanow et al., 2021). The clinical diagnosis of PD is based on motor symptoms such as bradykinesia, resting tremor, stiffness, and postural instability. The transition from prodromal PD to PD is characterized by the change from non-motor symptoms to clinically diagnosable motor symptoms (Meles et al., 2021). Olfactory dysfunction is one of the earliest non-motor features of PD, appearing several years before the onset of motor symptoms, and can predict the transition from prodromal PD to PD, independent of medication and age of onset (Tissingh et al., 2001; Dan et al., 2021). Therefore, olfactory neuroimaging can help to identify PD-specific olfactory neural phenotypes early and function as a precursive biomarker of the disease.

### Olfactory Disorders in Patients With Parkinson’s Disease

Patients with PD will have a series of motor symptoms, manifested as static tremor, bradykinesia, limb stiffness, and abnormal posture. Additionally, many non-motor symptoms also significantly affect the quality of life of patients with PD, such as olfactory disorders, constipation, depression, sleep disorders, and mild cognitive impairment (Doty, 2012; Aarsland and Kramberger, 2015). Braak et al. (2003) indicated that before presenting with motor symptoms, PD will involve the pre-olfactory nucleus and the olfactory bulb, which will cause olfactory disorders. Johnson and Ansari found that hyposmia was one of the more common non-motor symptoms in patients with PD, and appeared before the motor symptoms (Ansari and Johnson, 1975). Relevant studies have confirmed that about 45.90% of patients will experience varying degrees of hyposmia—or even loss of smell—3 years before the onset of motor symptoms (Haehner et al., 2009). Rolheiser et al. (2011) used Diffusion Tensor Imaging (DTI) to detect PD in patients and found that the diffusion range of the olfactory tract of the patients was significantly reduced compared to normal people. Some researchers have pointed out that the olfactory dysfunction of PD patients plays an especially important role in predicting the progression of the disease (Takeda et al., 2014).

To date, researchers have disputed the pathogenesis of PD olfactory dysfunction and have still not reached a consensus. Braak et al. (2003) postulated that the degeneration of the olfactory bulb and anterior olfactory nucleus may lead to olfactory dysfunction. Descarries et al. (1987) reported that a certain amount of DA neurotransmitters and DA receptors are stored in the piriform lobe, which is innervated by dopaminergic nerve fibers in the ventral tegmental area of the midbrain and the substantia nigra, indicating Dopaminergic nerve fiber connections exist between the olfactory system and the substantia nigra. It is speculated that when the dopaminergic nerve fibers that connect the olfactory system and the substantia nigra are damaged, it may lead to a decline in the olfactory function of PD patients. Additionally, some studies (Sengoku et al., 2008; Jellinger, 2009) have found that patients with PD have Lewy bodies clusters in the olfactory bulb and other olfactory-related brain areas such as the anterior olfactory nucleus, piriform cortex, amygdala, anterior olfactory cortex, and hippocampus. As the olfactory pathway progresses from the surrounding olfactory structure to the central olfactory bulb nucleus, it is confirmed that olfactory disorders may be related to the pathological changes of α-synuclein, and it is postulated that the degeneration of these regions causes changes in the olfactory function of PD patients.

### Neuroimaging Changes in Olfactory Disorders

Functional magnetic resonance imaging is a non-invasive imaging technique that can measure BOLD responses to task-induced or spontaneous neural activity. The latter is often called resting-state fMRI, and it is usually used to study the functional connections between different brain regions or networks. fMRI has been widely used in cognitive and sensory research—including smell—and many studies have used fMRI to determine the neural basis of both health and olfactory dysfunction (Pellegrino et al., 2016; Donoshita et al., 2021). As research increasingly focuses on the abnormal olfactory function associated with PD, there are increasing MRI studies regarding PD olfactory dysfunction, including the study of structural changes in the olfactory pathway and MRI functional imaging studies.

### Changes of Olfactory-Related Network in Parkinson’s Disease

Recently, research using resting-state fMRI in PD has become more extensive, from functional connection to regional homogeneity. Independent component analysis (ICA) of resting-state fMRI can identify several specific brain networks in both awake and resting states: default mode, saliency, and executive network (Wang et al., 2012, 2013, 2015a,b, 2017; Shi et al., 2015, 2017, 2018; Wu et al., 2020) and they have been used in a variety of neurodegenerative diseases including and have been extensively studied in a variety of neurodegenerative diseases—including PD (Pellegrino et al., 2016; Chang et al., 2017). Anatomically, the default mode network includes the medial prefrontal and posterior cingulate cortex. The significant network consists of the insula and the anterior cingulate cortices, and the central executive network includes the dorsolateral prefrontal and posterior parietal cortices. Functionally, the default mode network is associated with self-directed mental activity, usually inactive during task-induced fMRI, while the saliency network participates in the detection and filtering of external stimuli and internal brain events. The central executive network is usually activated together with the saliency network when resting and performing tasks. It plays a vital role in working memory, problem solving, and decision-making. Resting-state fMRI studies have confirmed that the functional coupling between these three networks changes in PD patients (Putcha et al., 2016).

Georgiopoulos et al. (2019) identified an olfactory network composed of posterior piriform cortex, insula, orbitofrontal cortex, and thalamus by including ICA data analysis based on time series and functional connection probability within the olfactory network; however, this network recruitment is not obvious in PD patients. The correlation between decreased fractional anisotropy (FA) and olfactory impairment in Parkinson’s disease patients located in the major olfactory cortical networks (OCN) and the direct gyrus indicates a conditional change in olfactory network connectivity. Central anosmia is a potential marker of early prodromal symptoms and disease progression, such as Parkinson’s disease and Alzheimer’s disease (Turetsky et al., 2009; Zou et al., 2015). Fjaeldstad et al. (2017) developed a new structural olfactory connection fingerprint identification method to study the olfactory cortex network, and constructed a combined OCN, which can be used as a potential neuroimaging biomarker for early structural changes in connections in certain disease states, such as Parkinson’s disease. We developed a new spectral contrast mapping (SCM) method to decode brain activity at the voxel level. Compared with healthy controls, there are differences in severe hyposmia in the frontal, parietal, and temporal gyrus. Therefore, the SCM can capture the abnormal neuron energy difference between patients with severe, no/mild hyposmia, and is a biomarker that reflects the state of the brain related to the sense of smell (Yu et al., 2021).

Although some progress has been made in the study of brain structure and functional magnetic resonance in Parkinson’s disease, little attention has been paid to the changes in olfactory-related white and gray matter connectivity. Increasingly, evidence shows that the BOLD signal in white matter undergoes stimulus-related synchronous changes after olfactory stimulation, and the signal changes in the white matter pathways of the brain show a certain degree of specificity (Ding et al., 2018). Our findings suggest that the dorsolateral prefrontal cortex, anterior entorhinal cortex, and fronto-orbital cortex in the gray matter of patients with Parkinson’s disease have abnormal connections with the posterior and superior corona radiata in the white matter. This may help in-depth study of the early pathological process of Parkinson’s disease.

### Functional Connectivity Changes in Olfactory-Related Areas in Parkinson’s Disease

Studies have shown that PD can damage a variety of resting state networks, such as movement, striatum, margins, and sensorimotor areas (Tahmasian et al., 2017; de Schipper et al., 2018). Additionally, changes in functional connectivity patterns are related to the severity of motor deficits (Shen et al., 2020), as well as to common non-motor symptoms in PD such as cognitive impairment and dementia (Weil et al., 2019), loss of smell (Yoneyama et al., 2018; Lee et al., 2020). The analysis of resting-state fMRI by Georgiopoulos et al. (2019) did not show any differences in smell, default mode, significance, or functional connectivity within the central executive network between Parkinson’s disease patients and the control group. They identified an olfactory network consisting of the posterior piriform cortex, insula, orbitofrontal cortex, and thalamus. The recruitment of this network in patients with PD is not obvious. GLM analysis shows that the bilateral insula and orbitofrontal cortex of patients with PD the activity is significantly reduced, but there is no significant difference in the olfactory cortex itself. Tremblay et al. (2020) and other studies found that the functional connection of PD patients in the chemosensory network was impaired, and no differences were found in the modularity of the identified network, but the network modularity of PD patients was significantly weakened when performing olfactory tasks. When performing trigeminal nerve tasks, no changes were found in patients with PD. Therefore, they postulated that the specific patterns of functional connectivity and chemosensory network recruitment in PD-related olfactory disorders can explain the different behavioral chemosensory characteristics in PD. The amygdala functional connectivity (FC) of PD patients with severe olfactory dysfunction is extensively reduced; especially the FC between the amygdala and subparietal lobules, lingual gyrus and fusiform gyrus, and it is related to the severity of hyposmia and cognitive ability (Yoneyama et al., 2018).

We utilized a white matter-gray matter FCC approach to provide a new perspective on Parkinson’s olfactory network connectivity. This approach could be used to find new potential brain regions that connect olfactory functions in Parkinson’s disease. We found that patients with Parkinson’s hyposmia in specific brain regions (dorsolateral prefrontal cortex, fronto-orbital cortex, anterior entorhinal cortex) have a special connection with the white matter fiber tracts (superior corona radiata, posterior corona radiata). The results showed that the abnormal functional covariance connection between the right hemisphere dorsolateral prefrontal cortex and white matter suggests that Parkinson’s patients have potential brain damage in this area. This indicator has clinical imaging value for the diagnosis of PD. The above-mentioned changes may be related to the up-regulation or down-regulation of olfactory chemoreceptor genes in the dorsolateral prefrontal cortex of Parkinson’s disease, resulting in clinical symptoms of hyposmia (Ansoleaga et al., 2015). The abnormal FCC between the dorsolateral prefrontal cortex and white matter may be due to the important role of neurons in the frontoorbital cortex in olfactory processing (Rolls and Baylis, 1994), and the specific mechanism needs further study.

### Weaknesses of This Study and Future Ideas

This study has several limitations. First, this study used image data from a public database. In order to study the brain function changes more accurately in Parkinson’s hyposmia, future research must construct and utilize our own unique image database. Second, we only used a FCC approach in resting-state fMRI to study alterations in olfactory-related functions. Future studies should utilize task-state and multimodal techniques to collect more comprehensive information such as functional connectivity or effective connectivity of olfactory stimulus dynamics in fMRI, cortical thickness, or diffusion tensor imaging in structural MRI. Third, our study did not obtain a comprehensive neuropsychological assessment, hindering our exploration of specific PD dysfunctions. In future work, we will recruit a larger sample to assess the neuropsychological status of PD patients more comprehensively. Finally, current cross-sectional studies cannot show causality, and we need to design further clinical trials to explore effective connectivity to reveal the neurophysiological mechanisms of PD in future.

## Conclusion

Early identification and diagnosis of olfactory disorders in patients with PD can help patients gain earlier access to treatment, and possibly delay the progression of the disease. Resting fMRI studies have shown that the impaired olfactory function in patients with early PD is related to abnormal changes in olfactory-related central structures. Our results show that the brain regions of the dorsolateral prefrontal, anterior entorhinal cortex and fronto-orbital cortices in gray matter have significant differences in functional covariance connections with the voxles in white matter. The function covariance connection strength of the right dorsolateral prefrontal cortex and white matter, and the covariance connection strength of the left superior corona radiata and gray matter function have potential diagnostic value. These may reflect the change of olfactory function in the early stage of PD, and could be a potential neuroimaging marker for early diagnosis.

## Data Availability Statement

Publicly available datasets were analyzed in this study. This data can be found here: The data was obtained from the OpenfMRI database. Its login number is ds000245 (https://www.openfmri.org/dataset/ds000245/). Detailed demographic data can be found in the original article (Yoneyama et al., 2018). The code can be obtained by contacting corresponding author of this article.

## Ethics Statement

The studies involving human participants were reviewed and approved by the Nagoya University Graduate School of Medicine. The ethics committee waived the requirement of written informed consent for participation.

## Author Contributions

ZC and NW played a critical role in conceptualizing this study. YQW, HW, and SD collected and analyzed all the data. YQW and HY contributed to writing the first draft of the manuscript. XL, YJW, JZ, and YW prepared the manuscript. All authors read and approved the submitted manuscript and provided critical feedback on the manuscript.

## Conflict of Interest

The authors declare that the research was conducted in the absence of any commercial or financial relationships that could be construed as a potential conflict of interest.

## Publisher’s Note

All claims expressed in this article are solely those of the authors and do not necessarily represent those of their affiliated organizations, or those of the publisher, the editors and the reviewers. Any product that may be evaluated in this article, or claim that may be made by its manufacturer, is not guaranteed or endorsed by the publisher.

## Footnotes

1
https://www.openfmri.org/dataset/ds000245/

2
http://rfmri.org/dpabi

3
https://www.fil.ion.ucl.ac.uk/spm/

4
https://www.nitrc.org/projects/mricron

5
https://ww2.mathworks.cn/
